# Alcohol Use and Related Problems Along the United States–Mexico Border

**DOI:** 10.35946/arcr.v38.1.10

**Published:** 2016

**Authors:** Britain A. Mills, Raul Caetano

**Affiliations:** Britain A. Mills, Ph.D., is a research associate at the University of Texas School of Public Health, Dallas Regional Campus, Dallas, Texas. Raul Caetano, M.D., Ph.D., is Dean University of Texas Southwestern School of Health Professions, and Regional Dean, Dallas Regional Campus, University of Texas School of Public Health, Dallas, Texas

The southern border the United States shares with Mexico has been of particular interest to alcohol researchers because of the presence of multiple risk factors conducive to alcohol- related problems. The border region spans 2,000 miles and is home to more than 7 million U.S. residents of predominantly Mexican-American ethnicity.

Compared with other areas of the United States, border residents have higher rates of poverty, undereducation, and unemployment (Gerber 2009; Soden 2006). They also are at elevated risk for multiple negative health outcomes, including tuberculosis, hepatitis A, diabetes, and liver disease (Centers for Disease Control and Prevention 2008*a*,*b*; Pan American Health Organization 2007; Texas Comptroller of Public Accounts 2003) and are differentially affected by crime related to illegal drug trafficking (Office of National Drug Control Policy 2011).

The border also separates two distinct geopolitical areas with longstanding differences in alcohol policy. In Mexico, the legal drinking age is 18, compared with 21 in the United States, and alcohol is comparatively inexpensive. The many Mexican bars within walking distance of the border cater primarily to people in younger age-groups who travel from U.S. border towns to Mexico specifically to capitalize on the cheap alcohol and easier access (Lange and Voas 2000; Lange et al. 2002).

Consistent with the risk factors described above, early studies of alcohol use within border populations showed that border residents were at higher risk for some alcohol outcomes compared with people who do not live near the border. However, the findings varied depending on the following factors:

- The populations studied—for example, Texas versus California;
- The comparison group used—for example, U.S. Hispanics versus U.S. Mexican Americans; and
- The specific alcohol outcome in question—for example, alcohol use versus alcohol-related problems (Substance Abuse and Mental Health Services Administration 2004; Wallisch 1998; Wallisch and Spence 2006; see also Harrison and Kennedy 1996; Holck et al. 1984).

Demonstrating the difficulties of finding good comparison groups, one study (Wallisch and Spence 2006) showed that, compared with more densely populated areas, rates of binge drinking and alcohol dependence tend to be higher in colonias, which are unregulated and sparsely populated settlements within the U.S. border region that often lack basic public services.

In more recent studies, researchers have drawn samples from geographic areas spanning the entire border region, and they have shifted the focus to comparisons between more ethnically homogeneous subgroups on and off the border, with the goal of clarifying the precise risk conferred by living in the border region. In general, these studies find that drinking levels are higher in U.S. border regions, regardless of ethnicity, compared with non-border regions and are particularly elevated among younger age-groups (Caetano et al. 2012; Liu 2012). Similar patterns are seen for alcohol-problem outcomes such as abuse, dependence, and social problems (Caetano et al. 2013*c*; Vaeth et al. 2012). Despite these findings of generally higher levels of alcohol use and related problems, in general, rates of driving under the influence do not differ on and away from the border (Caetano et al. 2013*b*), and border residents do not report more treatment seeking for alcohol-related problems than non–border residents (Reingle et al. 2014). Both findings, however, are consistent with risks that primarily are restricted to younger age-groups in the region, particularly considering that younger age-groups have not had time to consume large cumulative quantities of alcohol that lead to chronic alcohol problems and typically precede treatment seeking.

One factor that clearly contributes to elevated alcohol-related risks along the U.S. side of the border is the ability to temporarily cross into Mexico to drink. This leads to generally higher annual levels of drinking and alcohol-related problems on the U.S. side of the border, particularly among younger age-groups who deliberately exploit Mexico’s lower legal drinking age. For example, among current drinkers living on the U.S. side of the border, those who reported any drinking in Mexico in the past year tended to be younger and reported significantly more alcohol intake (measured in volume), higher rates of binge drinking, and higher rates of alcohol problems than those who reported drinking only in the United States (Caetano et al. 2013*a*; Clapp et al. 2001). Many of these individuals cross the border on foot, spend the evening patronizing the local bars, and return to their cars on the U.S. side in the early hours of the morning (Lange and Voas 2000). When bars in the border city of Juárez, Mexico, shifted to an earlier closing time (from 5 a.m. to 2 a.m.), the percentage of people crossing back into the United States with a positive blood alcohol content dropped by 89 percent (Voas et al. 2002). A second factor associated with higher alcohol-related risks among U.S. border residents seems to be drinking in bars, as opposed to elsewhere, whether on the Mexico side or the U.S. side of the border. Among U.S. border residents, more than 75 percent report not traveling to Mexico at all in the past year, and young adult border residents report more drinking than other groups, regardless of whether they cross into Mexico to drink (Caetano et al. 2012, 2013*a*). Surprisingly, young adult border residents who reported not traveling to Mexico to drink actually reported slightly higher rates of past-year bar attendance (75 percent) than those who reported drinking in Mexico (69 percent), both of which were higher than rates of past-year bar attendance among non-border young adults (59 percent). Moreover, the specific pattern of differences on and off the border in drinking (Mills et al. 2012, 2014) and acute alcohol problems are precisely mirrored in, and are statistically explained by, patterns of bar attendance across these areas. These effects cannot be attributed to age or border/non-border differences in the ways people think about drinking (e.g., more liberal drinking attitudes) or perceptions of broad neighborhood characteristics (e.g., perceptions of violence). Bar attendance seems to be a key contributing factor to elevated alcohol-related risks among the border region’s younger population. Therefore, future research would benefit from identifying characteristics of these on-premise alcohol outlets in border areas, including their geographic distribution (Berke el at. 2010; Pollack et al. 2005; Romley et al. 2007) and characteristics of their clientele (Graham et al. 2006).

In sum, U.S. residents living near the country’s border with Mexico are at higher risk for alcohol use and related consequences. This risk is accentuated among young people and is tightly connected to this group’s higher frequency of bar attendance, whether on the U.S. or Mexico side of the border. Travelling to Mexico to drink— a major focus of early border research—contributes to this risk but falls short of fully explaining it. U.S. policymakers should be aware that high levels of alcohol-related risks on the border are not simply a south-of-the-border phenomenon. To a large extent, they reflect factors within U.S. borders that are under their direct control.

## References

[b1-arcr-38-1-79] Berke EK, Tanski SE, Demidenko E (2010). Alcohol retail density and demographic predictors of health disparities: A geographic analysis. American Journal of Public Health.

[b2-arcr-38-1-79] Caetano RC, Mills BA, Vaeth PAC (2012). Alcohol consumption and binge drinking among U.S.-Mexico border and non-border Mexican Americans. Alcoholism: Clinical and Experimental Research.

[b3-arcr-38-1-79] Caetano RC, Mills BA, Vaeth PAC (2013a). Alcohol use among Mexican American U.S.–Mexico border residents: Differences between those who drink and who do not drink in Mexico. Addictive Behaviors.

[b4-arcr-38-1-79] Caetano RC, Vaeth PAC, Mills BA (2013b). Rates and predictors of DUI among U.S.–Mexico border and non-border Mexican Americans. Accident Analysis and Prevention.

[b5-arcr-38-1-79] Caetano RC, Vaeth PAC, Mills BA, Rodriguez LA (2013c). Alcohol abuse and dependence among U.S.–Mexico Border and Non-Border Mexican Americans. Alcoholism: Clinical and Experimental Research.

[b6-arcr-38-1-79] Centers for Disease Control and Prevention (CDC) (2008a). Morbidity Trend Tables United States: Tuberculosis Cases.

[b7-arcr-38-1-79] Centers for Disease Control and Prevention (CDC) (2008b). Reported Cases of Acute Viral Hepatitis, by Type and Year, United States, 1966–2003.

[b8-arcr-38-1-79] Clapp JD, Voas RB, Lange JE (2001). Cross-border college drinking. Journal of Safety Research.

[b9-arcr-38-1-79] Gerber J (2009). Developing the U.S.-Mexico Border Region for a Prosperous and Secure Relationship: Human and Physical Infrastructure Along the U.S. Border With Mexico.

[b10-arcr-38-1-79] Graham K, Bernards S, Osgood DW, Wells S (2006). Bad nights or bad bars? Multi-level analysis of environmental predictors of aggression in late-night large-capacity bars and clubs. Addiction.

[b11-arcr-38-1-79] Harrison LD, Kennedy NJ (1996). Drug use in the high intensity drug trafficking area of the US Southwest border. Addiction.

[b12-arcr-38-1-79] Holck SE, Warren CW, Smith JC, Rochat RW (1984). Alcohol consumption among Mexican American and Anglo women: Results of a survey along the U.S.-Mexico border. Journal of Studies on Alcohol.

[b13-arcr-38-1-79] Lange JE, Voas RB (2000). Youth escaping limits on drinking: Binging in Mexico. Addiction.

[b14-arcr-38-1-79] Lange JE, Voas RB, Johnson MB (2002). South of the border: A legal haven for underage drinking. Addiction.

[b15-arcr-38-1-79] Liu LY (2012). 2010 Texas School Survey of Substance Use Among Students: Grades 7–12.

[b16-arcr-38-1-79] Mills BA, Caetano RC, Vaeth PAC (2012). What explains higher levels of drinking among Mexican Americans on the U.S-Mexico border?. Alcoholism: Clinical and Experimental Research.

[b17-arcr-38-1-79] Mills BA, Caetano R, Vaeth PAC (2014). Crossborder policy effects on alcohol outcomes: Drinking without thinking on the U.S.-Mexico border?. Alcoholism: Clinical and Experimental Research.

[b18-arcr-38-1-79] Office of National Drug Control Policy (2011). High Intensity Drug Trafficking Areas (HIDTA) Program: Report to Congress.

[b19-arcr-38-1-79] Pan American Health Organization (PAHO) (2007). United States-Mexico border area. Health in Americas, Volume II—Countries.

[b20-arcr-38-1-79] Pollack CE, Cubbin C, Ahn D, Winkleby M (2005). Neighbourhood deprivation and alcohol consumption: Does the availability of alcohol play a role?. International Journal of Epidemiology.

[b21-arcr-38-1-79] Reingle Gonzalez JM, Caetano R, Mills BA, Vaeth PA (2014). An assessment of individual-level factors associated with alcohol treatment utilization among Mexican Americans. Journal of Substance Abuse Treatment.

[b22-arcr-38-1-79] Romley JA, Cohen D, Ringel J, Strum R (2007). Alcohol and environmental justice: The density of liquor stores and bars in urban neighborhoods in the United States. Journal of Studies on Alcohol and Drugs.

[b23-arcr-38-1-79] Soden DJ (2006). At the Cross Roads: US/Mexico Border Countries in Transition.

[b24-arcr-38-1-79] Substance Abuse and Mental Health Services Administration (SAMHSA) (2004). Alcohol Dependence or Abuse and Age at First Use.

[b25-arcr-38-1-79] Texas Comptroller of Public Accounts (2003). The Border: Snapshot.

[b26-arcr-38-1-79] Vaeth PAC, Caetano R, Mills BA, Rodriguez LA (2012). Alcohol-related social problems among Mexican Americans living in U.S.-Mexico border and non- border areas. Addictive Behaviors.

[b27-arcr-38-1-79] Voas RB, Lange JE, Johnson MB (2002). Reducing high-risk drinking by young Americans south of the border: The impact of a partial ban on sales of alcohol. Journal of Studies on Alcohol.

[b28-arcr-38-1-79] Wallisch LS (1998). The 1996 Survey of Substance Use on the Texas-Mexico Border and in Colonias.

[b29-arcr-38-1-79] Wallisch LS, Spence RT (2006). Alcohol and drug use, abuse, and dependence in urban areas and colonias of the Texas-Mexico border. Hispanic Journal of Behavioral Sciences.

